# Assessing degradation of composite resin cements during artificial aging by Martens hardness

**DOI:** 10.1186/s13005-017-0142-4

**Published:** 2017-05-19

**Authors:** Stefan Bürgin, Nadja Rohr, Jens Fischer

**Affiliations:** Division of Dental Materials and Engineering, Department of Reconstructive Dentistry and Temporomandibular Disorders, University Center for Dental Medicine Basel, University of Basel, Hebelstrasse 3, 4056 Basel, Switzerland

**Keywords:** Resin composite cement, Martens hardness, Indirect tensile strength, Aging, Thermal cycling

## Abstract

**Background:**

Aim of the study was to verify the efficiency of Martens hardness measurements in detecting the degradation of composite resin cements during artificial aging.

**Methods:**

Four cements were used: Variolink II (VL2), RelyX Unicem 2 Automix (RUN), PermaFlo DC (PDC), and DuoCem (DCM). Specimens for Martens hardness measurements were light-cured and stored in water at 37 °C for 1 day to allow complete polymerization (baseline). Subsequently the specimens were artificially aged by water storage at 37 °C or thermal cycling (*n* = 6). Hardness was measured at baseline as well as after 1, 4, 9 and 16 days of aging. Specimens for indirect tensile strength measurements were produced in a similar manner. Indirect tensile strength was measured at baseline and after 16 days of aging (*n* = 10). The results were statistically analyzed using one-way ANOVA (α = 0.05).

**Results:**

After water storage for 16 days hardness was significantly reduced for VL2, RUN and DCM while hardness of PDC as well as indirect tensile strength of all cements were not significantly affected. Thermal cycling significantly reduced both, hardness and indirect tensile strength for all cements. No general correlation was found between Martens hardness and indirect tensile strength. However, when each material was analyzed separately, relative change of hardness and of indirect tensile strength revealed a strong linear correlation.

**Conclusions:**

Martens hardness is a sensible test method to assess aging of resin composite cements during thermal cycling that is easy to perform.

## Background

Advances in CAD/CAM technology have increased the application of all-ceramic restorations in dentistry [1, 2]. In clinical use, ceramic has proven to be a reliable restorative material showing survival rates of up to 93% after 10 years [3–8]. Ceramic materials provide high aesthetic options but they are susceptible to tensile stress, which implies a higher risk of failures during functional loading, such as veneer chipping or fractures [9–11]. To prevent any tensile stress in the ceramic, the restoration should not exhibit mechanical retention on the prepared tooth [12]. Therefore, a stable adhesive cementation is decisive for the long term success of ceramic restorations [13]. Further, it has been shown that the strength of ceramic restorations is doubled when adhesively cemented, because the intaglio surface of the restoration is shielded from tensile stress [14].

Adhesive cementation is technique-sensitive and requires several steps. The hydrophilic tooth surface must be conditioned in order to remove the smear layer and to create a micro-structured surface. In a second step the tooth surface must be primed in order to make it wettable to a hydrophobic resin composite cement. Priming is accomplished by bifunctional monomers in the primer such as HEMA, which are applied at first and bind to the tooth substance with a hydrophilic end while the hydrophobic end contains a polymerizable group that can bind to the resin composite cement [15].

Efforts were made to simplify the process of surface conditioning for adhesive cementation [16]. Therefore, besides the traditional "etch and rinse" systems, which require all steps of surface treatment, self-adhesive cements were developed, comprising monomers with phosphate groups, which are able to chemically bond to the tooth substance.

In the oral cavity restorations are subjected to mechanical, chemical and thermal stress and thus undergo a process of aging. To simulate the effect of the humid environment in the oral cavity by laboratory testing, storage in water or artificial saliva at 37 °C may be performed. To simulate the effect of temperature changes in the oral cavity, thermal cycling is usually applied [17–20]. Specimens are in turn immersed in water of 5 °C and 55 °C, simulating extreme situations with exposure to ice cream and hot beverages.

To analyze the effect of aging, specimens for flexural strength test can be used [21]. But the preparation of these specimens is time consuming and complex. An alternative is to use the indirect tensile strength test [22–25]. In contrast to flexural specimens the preparation of indirect tensile specimens is less complex. However, as any chemical stress on restorative materials generated in the oral cavity particularly affects the superficial layers of the respective material, assessment of aging effects might also be achieved by hardness measurement, which is a very easily performed test. The only requirement is an even and smooth surface of the specimen. A further advantage of hardness measurements is that multiple measurements can be performed on one specimen, thus alterations over time can be followed.

Published results investigating correlations between strength and hardness of resin composite materials are not consistent. In various studies it has been demonstrated that hardness of composite filling materials is not affected by thermal cycling, while flexural strength decreases significantly under the same conditions [26–29]. In contrast to these findings Medeiros et al. [24] report on a positive correlation between Vickers hardness and indirect tensile strength when investigating the aging of a composite resin restorative material, while Aguiar et al. [25] observed the opposite. However, they both used micro-hardness, which measures only the most superficial layer of the specimens and all measurements were performed after only 24 h water storage. Further, it is unknown whether these observations also apply for resin composite cements.

In all studies Vickers hardness was used. Compared to other hardness test methods, Martens hardness has the advantage that not only plastic but also elastic deformation is measured, which is closer to flexural or indirect tensile strength measurements. Hence, the aim of this study was to analyze the efficiency of Martens hardness measurements in assessing the effect of artificial aging on resin composite cements. Previous investigations with one cement have shown that 16 days of aging by water storage or thermal cycling are sufficient to detect aging effects [23]. Therefore, hardness measurements were performed after water storage at 37 °C or thermal cycling up to 16 days. Indirect tensile strength tests before aging and after 16 days of aging were conducted for the reason of comparison.

## Methods

### Cements

Four different resin composite cements were used in this study, three conventional multi-step materials and one self-adhesive cement (Table 1).

**Table 1:** Summary of cements used

Name	Abbreviation	Manufacturer	Composition	Mixing mode
Variolink II	VL2	Ivoclar Vivadent, Schaan, Liechtenstein	Matrix: Bis-GMA, urethane dimethacrylate, triethylene glycol dimethacrylate Fillers: barium glass, ytterbium trifluoride, Ba-Al-fluorosilicate glass, spheroid mixed oxide	hand mixing
RelyX Unicem 2 Automix	RUN	3 M ESPE, Seefeld, Germany	Matrix: methacrylate monomers, methacrylated phosphoric esters, initiator components, stabilizers, rheological additives, pigments, Fillers: alkaline (basic) fillers, silanated fillers	automix tip
PermaFlo DC	PDC	Ultradent Products, Jordan, Utah, USA	Matrix: Bis-GMA, triethylene glycol dimethacrylate Fillers: no indications	automix tip
DuoCem	DCM	Coltène/Whaledent, Altstätten, Switzerland	Matrix: methacrylate monomers Fillers: barium glass, amorphous silic acid, fluoride	automix tip

### Specimen preparation for Martens hardness measurements

To measure Martens hardness, specimens with dimensions of 25.0 × 2.0 × 2.0 mm were produced in sets of 6 as described in ISO 4049. The cements were mixed according to the manufacturer’s instructions and filled into cavities of a customized Teflon mold. Glass slabs 1 mm in thickness and covered with a transparent Mylar foil (Hawe Transparent Strips, KerrHawe, Bioggio, Switzerland) were placed on both sides of the mold to keep the cement in place.

Light-curing was performed with a polymerization lamp (Elipar, 3 M ESPE, Seefeld, Germany) providing an intensity of 1200 mW/cm^2^. The exit window of the light-curing device had a diameter of 8 mm. It was positioned directly on the glass slab, which means in a well-defined distance of 1 mm to the specimen’s surface. Light-curing was started in the center of the specimen. After light exposure the exit window was moved to the section next to the center overlapping the previous section by half the diameter of the exit window (i.e. 4 mm). The procedure was reiterated until the specimen on the one side of the center had been completely exposed to the light. Thereafter the section on the other side of the center was light-cured in the same manner. The whole procedure was repeated from the rear side of the specimen. Duration of each light exposure was 10 s (PDF, RUN, and VL2) or 20 s (DCM) according to the manufacturers’ instructions.

### Specimen preparation for indirect tensile strength tests

To measure indirect tensile strength, cylindrical test specimens (3 mm in height and 3 mm in diameter) were produced in sets of 10 using a customized Teflon mold. The cements were mixed according to the manufacturer’s instructions and filled into the cavities of the Teflon mold. A glass slab covered with a transparent Mylar foil was pressed on each side of the mold and kept in place with a clamp. Each specimen was light-cured on both sides for 20 s. After demolding excess material was carefully removed.

### Measurement of Martens hardness

Martens hardness was measured with a universal testing machine (ZHU 2.5, Zwick/Roell, Ulm, Germany), using a Vickers indenter. A load of 10 N was applied. The crosshead speed was set to 5 N/min. Each hardness value was calculated as mean of three indentations. Furthermore, plastic and elastic indentation work was registered.

### Measurement of indirect tensile strength

To measure indirect tensile strength, specimens were loaded in a universal testing machine (Z020, Zwick/Roell, Ulm, Germany) perpendicular to the cylinder axis until fracture. The crosshead speed was set to 1 mm/min. The indirect tensile strength was calculated according to the following equation:

$$ \upsigma = 2\mathrm{F}/\uppi \mathrm{d}\mathrm{h} $$

F = load at fracture; d = diameter of the specimen; h = height of the specimen

### Endpoint of post-polymerization

To identify the endpoint of post-polymerization after light-curing, specimens for hardness measurement were prepared (*n* = 6). The specimens were removed from the mold immediately after light-curing and Martens hardness was measured. Subsequently the specimens were stored in deionized water at 37 °C. Martens hardness was measured on all specimens after 1, 6, 12, 24, 48 and 96 h storage time.

### Artificial aging

From each cement 2 sets of specimens for Martens hardness measurements were prepared. On all specimens hardness was measured immediately after light-curing. Subsequently, the specimens were stored in water for 1 day at 37 °C to allow complete polymerization and hardness was measured again prior to aging (baseline value).

Indirect tensile strength after light-curing was measured with 1 set of specimens. All further sets of specimens were stored in deionized water at 37 °C for 1 day to allow complete polymerization. Indirect tensile strength after 1 day of water storage was measured with 1 set of specimens (baseline value).

For each cement, 1 set of specimens for Martens hardness measurements and 1 set for indirect tensile strength tests were stored in deionized water at 37 °C, 1 set of specimens for Martens hardness measurements and 1 set for indirect tensile strength tests were thermo-cycled. For thermal cycling a customized device was used. The specimens were immersed alternately in water baths of 5 °C and 55 °C, using a sieve for storage and transportation. The cycle duration was 1 min with a dwell time of 28 s in each bath and a transfer time of 2 s between the baths.

Hardness was measured after 1, 4, 9, and 16 days of storage on the same specimens. Indirect tensile strength was measured after 16 days of aging. For PDC an additional value of indirect tensile strength after 4 days of thermal cycling was obtained with one more set of specimens.

### Statistics

All groups were tested for normal distribution with Shapiro-Wilk test. The groups were normal distributed and therefore compared using one-way analysis of variance (ANOVA, SPSS, SPSS Inc., Chicago, IL, US). All results with a *P*-value < 0.05 were considered as statistically significantly different.

## Results

### Endpoint of post-polymerization

**Fig. 1:**
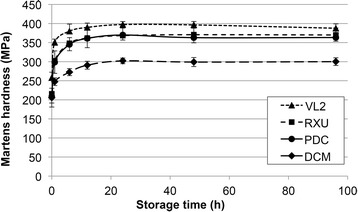
Martens hardness as a function of time during post-polymerization

In the first 24 h of water storage an increase in hardness was found in all 4 cements. Then hardness remained constant (Fig. 1).

### Effect of aging

**Fig. 2:**
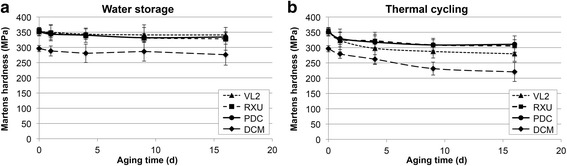
Martens hardness as a function of time during **a** water storage **b** thermal cycling

Short-term water storage for 16 days slightly affected Martens hardness (Fig. 2a). After thermal cycling the effect was more pronounced and already evident after 1 day of aging (Fig. 2b).

**Fig. 3:**
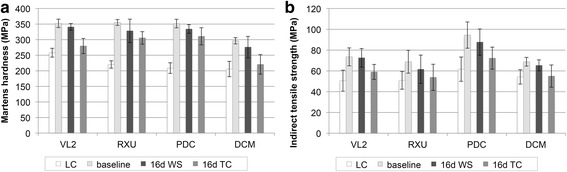
Means and standard deviations of **a** Martens hardness and **b** indirect tensile strength after light-curing (LC), at baseline, after 16 days of water storage (WS) and after 16 days of thermal cycling (TC)

Figure 3a and b are provided to compare the means and standard deviations of Martens hardness and indirect tensile strength, respectively, after light-curing, at baseline (1 day water storage at 37 °C), after 16 days aging by water storage at 37 °C and after 16 days aging by thermal cycling.

In all cases baseline values for hardness and indirect tensile strength were significantly higher than after light-curing (*p* < 0.001). After water storage for 16 days hardness was significantly reduced for VL2 (*p* = 0.008), RUN (*p* = 0.006), DCM (*p* = 0.022) and PDC (*p* < 0.001) while indirect tensile strength of all cements were not significantly affected (*p* > 0.05). Thermal cycling for 16 days in all cases significantly reduced hardness and indirect tensile strength in comparison to the respective baseline values (*p* < 0.01).

**Fig. 4:**
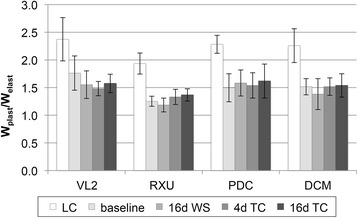
Ratio of plastic and elastic indentation work

The ratio of the plastic and elastic indentation work significantly dropped after complete polymerization, compared to the situation directly after light-curing, but no further change was observed during aging (Fig. 4).

### Correlation between Martens hardness and indirect tensile strength

**Fig. 5:**
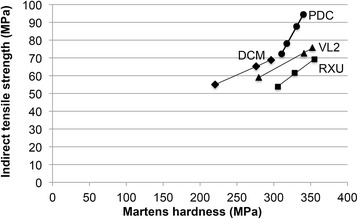
Correlation between Martens hardness and indirect tensile strength for each cement

**Fig. 6:**
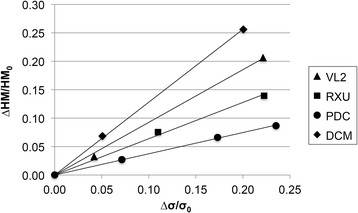
Correlation between relative change of Martens hardness and relative change of indirect tensile strength for each cement

Although the patterns of Martens hardness (Fig. 3a) and indirect tensile strength (Fig. 3b) are similar, no general correlation between both values was found. However, a strong linear correlation was observed for each particular material (Fig. 5). In addition, when plotting the relative change of Martens hardness (absolute decrease of Martens hardness [ΔHM] divided by the baseline value [HM_0_]) against the relative change of indirect tensile strength (absolute decrease of indirect tensile strength [Δσ] divided by the baseline value [σ_0_]) a strong linear correlation is obvious with all 4 regression curves running through the origin (Fig. 6).

## Discussion

### Endpoint of post-polymerization

The obtained results when following the polymerization process after light-curing suggest that hardness measurements correlate with the degree of polymerization of the cements. To the knowledge of the authors the use of Martens hardness measurements in order to characterize the progress of polymerization is not published yet. However, the process of polymerization of resin cements was previously characterized using Knoop-hardness [30, 31]. Vickers [32, 33] and Knoop hardness [34] have been applied to test the degree of polymerization [32, 34] and polymerization shrinkage [33]. All 4 tested cements reached a constant level of hardness after 1 day of storage time, indicating that the polymerization was substantially finished. It was reasoned from the results that in a laboratory test setup a 1 day water storage at 37 °C is recommended for all 4 cements to allow complete polymerization.

### Artificial aging

In the present study the effect of short-term water storage at 37 °C on indirect tensile strength is not as effective as observed in a previous investigation, where 16 days of water storage significantly reduced the indirect tensile strength of a different cement [22]. Therefore, the immersion time in water over 16 days was too short to produce a measurable effect with Martens hardness.

In the present as well as in a previous study [22] thermal cycling revealed to be more effective. However, published results in general are not consistent. That may be explained to some extent by the various methods, which differ in the duration for one cycle and the temperature or the storing medium. Some authors determined a slight decrease [26, 29] while others found a slight increase in strength [27–29].

A specific correlation between Martens hardness and indirect tensile strength for each cement was observed in the present study. A study with a similar test design, which evaluated Vickers hardness and indirect tensile strength was published by Medeiros et al. [23]. Artificial aging of one light-cured composite resin restorative material was carried out by storing the specimens in alcohol, acetic acid, or propionic acid. Regression between mean values of Vickers hardness and indirect tensile strength reveal a linear correlation. Aguiar et al. [25] did not find any correlation between hardness and indirect tensile strength when investigating several different materials. Both results correspond to the present findings. For one single material a correlation between hardness and indirect tensile strength is given, but when comparing multiple materials, no general correlation can be found.

The most important finding in the present study is the fact that the relative variations of Martens hardness and indirect tensile strength following aging reveal a very strong linear correlation specific for each cement, with the regression line running exactly through the point of origin. However, a comparison between different materials must be performed carefully.

Aging in water resulted in significant differences between Martens hardness but not for indirect tensile strength values for all cements when compared to baseline. Therefore measuring Martens hardness can be considered a more sensitive test method for evaluating aging procedures of resin composite cements than indirect tensile strength test.

The invariable ratio between plastic and elastic indentation work during aging indicates that no structural changes in the materials such as an embrittlement occurred which could have changed elasticity or plasticity.

### Limitations of the study

It is proposed that 10,000 cycles may represent 1 year of service [18]. In the present study thermal cycling was performed over 16 days indicating more than 2 years of service. For water storage at 37 °C an immersion time of 16 days was too short to induce same degradations as they were found for specimens that were aged by thermal cycling. Martens hardness and indirect tensile strength measurements seem to be suitable methods to detect surface degradations induced by aging. However, further investigations are required to test the change in depth of the specimens over time.

## Conclusions

Within the limitations of this study it can be concluded that:

- For artificial aging of resin composite cements thermal cycling (5 °C/55 °C) is more effective than water storage at 37 °C.
- Degradation of resin composite cements after artificial aging may be detected by Martens hardness measurements.
- The relative changes in Martens hardness and indirect tensile strength induced by aging are strongly correlated within one material.

## Abbreviations

DCM: DuoCem
HM: Martens hardness
PDC: PermaFlo DC
RUN: RelyX Unicem 2 Automix
VL2: Variolink II
